# Modelling human placental villous development: designing cultures that reflect anatomy

**DOI:** 10.1007/s00018-022-04407-x

**Published:** 2022-06-26

**Authors:** Joanna L. James, Abbey Lissaman, Yohanes N. S. Nursalim, Lawrence W. Chamley

**Affiliations:** 1 Department of Obstetrics and Gynaecology, Faculty of Medical and Health Sciences, University of Auckland, Auckland, New Zealand; 2 Department of Physiology, Faculty of Medical and Health Sciences, University of Auckland, Auckland, New Zealand

**Keywords:** Placental development, In vitro models, Trophoblast stem cell, Extravillous trophoblast, Syncytiotrophoblast

## Abstract

The use of in vitro tools to study trophoblast differentiation and function is essential to improve understanding of normal and abnormal placental development. The relative accessibility of human placentae enables the use of primary trophoblasts and placental explants in a range of in vitro systems. Recent advances in stem cell models, three-dimensional organoid cultures, and organ-on-a-chip systems have further shed light on the complex microenvironment and cell–cell crosstalk involved in placental development. However, understanding each model’s strengths and limitations, and which in vivo aspects of human placentation in vitro data acquired does, or does not, accurately reflect, is key to interpret findings appropriately. To help researchers use and design anatomically accurate culture models, this review both outlines our current understanding of placental development, and critically considers the range of established and emerging culture models used to study this, with a focus on those derived from primary tissue.

## Introduction

The placenta is a critical fetal organ that facilitates the transport of gasses, nutrients and wastes between mother and baby to support fetal growth during pregnancy. However, despite its importance for pregnancy success, the development and function of the placenta itself is not well understood. The placenta is comprised of branching villi that continue to grow across gestation. Each villus has a core of mesenchymal cells, placental macrophages (Hofbauer cells) and fetal blood vessels. This core is surrounded by a specialised epithelial trophoblast bilayer consisting of proliferative mononuclear cytotrophoblast overlain by a multinucleated syncytiotrophoblast that is bathed in maternal blood for most of pregnancy. Together, the branching placental architecture, function of the trophoblast bilayer, and development of the blood vessels within the villi, enable efficient nutrient and gas uptake into the fetal circulation and are critical for healthy placental function.

Early placental development is highly coordinated, and disruptions to implantation or placentation in early pregnancy can lead to pregnancy disorders in later gestation such as preeclampsia and fetal growth restriction (FGR), which together complicate around one in ten pregnancies [1, 2]. Pre-eclampsia occurs in 3–5% of pregnancies, and is defined by new-onset hypertension (≥ 140 mmHg systolic; ≥ 90 mmHg diastolic) after 20 weeks gestation together with one or more of “proteinuria and/or evidence of maternal acute kidney injury, liver dysfunction, neurological features, hemolysis or thrombocytopenia, and/or fetal growth restriction” [3]. FGR occurs when fetal growth decelerates or stagnates in utero, meaning the fetus does not reach its growth potential [4]. Clinically, FGR is defined as babies born < 3rd growth percentile, and is a leading contributor to stillbirth [5]. FGR and pre-eclampsia both result from placental dysfunction, with distinct but overlapping pathophysiologies. As a result, approximately 30% of pre-eclamptic pregnancies also exhibit FGR [1]. The temporal disconnect between the pathophysiological origins of pre-eclampsia and FGR in early pregnancy, and their clinical appearance in the second half of gestation, makes making these disorders challenging to understand, predict, or treat, and better understanding of the complex molecular events that occur during early placental development is key to improve this.

Our ability to accurately model early human placental development is limited by the clear ethical restrictions that prevent direct access to intact implantation sites in vivo in very early pregnancy. Furthermore, whilst first trimester placental and decidual tissues can be obtained from pregnancy terminations, this resource is not universally available to all researchers, and our inability to predict pregnancy disorders in early pregnancy means it is not possible to distinguish normal and abnormal tissue at this stage of gestation. In contrast, term placentae obtained after delivery are one of the most accessible human tissues, but key phenotypic and functional differences exist in placental cells and villi across gestation [6]. Finally, whilst in vivo animal models of pregnancy are useful for understanding many features of pregnancy pathologies, key anatomical differences with respect to blastocyst implantation, the extent of trophoblast invasion into, and remodelling of the decidua, and the distinct human trophoblast lineages formed, mean significant limitations remain in their use to understand early human placental development [7].

As a result of the above, the mainstay of placental research has for many years been based in relatively short-term cultures of primary tissue (explants or isolated trophoblasts), or transformed cell lines, both of which have their own sets of advantages and disadvantages. In recent years advances in in vitro culture techniques, including improved stem cell models, three-dimensional (3D) organoid cultures, and organ-on-a-chip models that better recapitulate the complex microenvironment and cellular cross-talk involved in placental development have emerged. Regardless of the model chosen, understanding each model’s strengths and weaknesses and which in vivo aspects of human placentation study findings do, or do not, accurately reflect, is key to interpret data appropriately. This review aims to consider the range of established and emerging culture models to study placental development, with a focus on those derived from primary tissue, to help researchers use and design culture models that accurately reflect the various anatomical aspects of placental development being studied.

### Early placental development

**Fig. 1:**
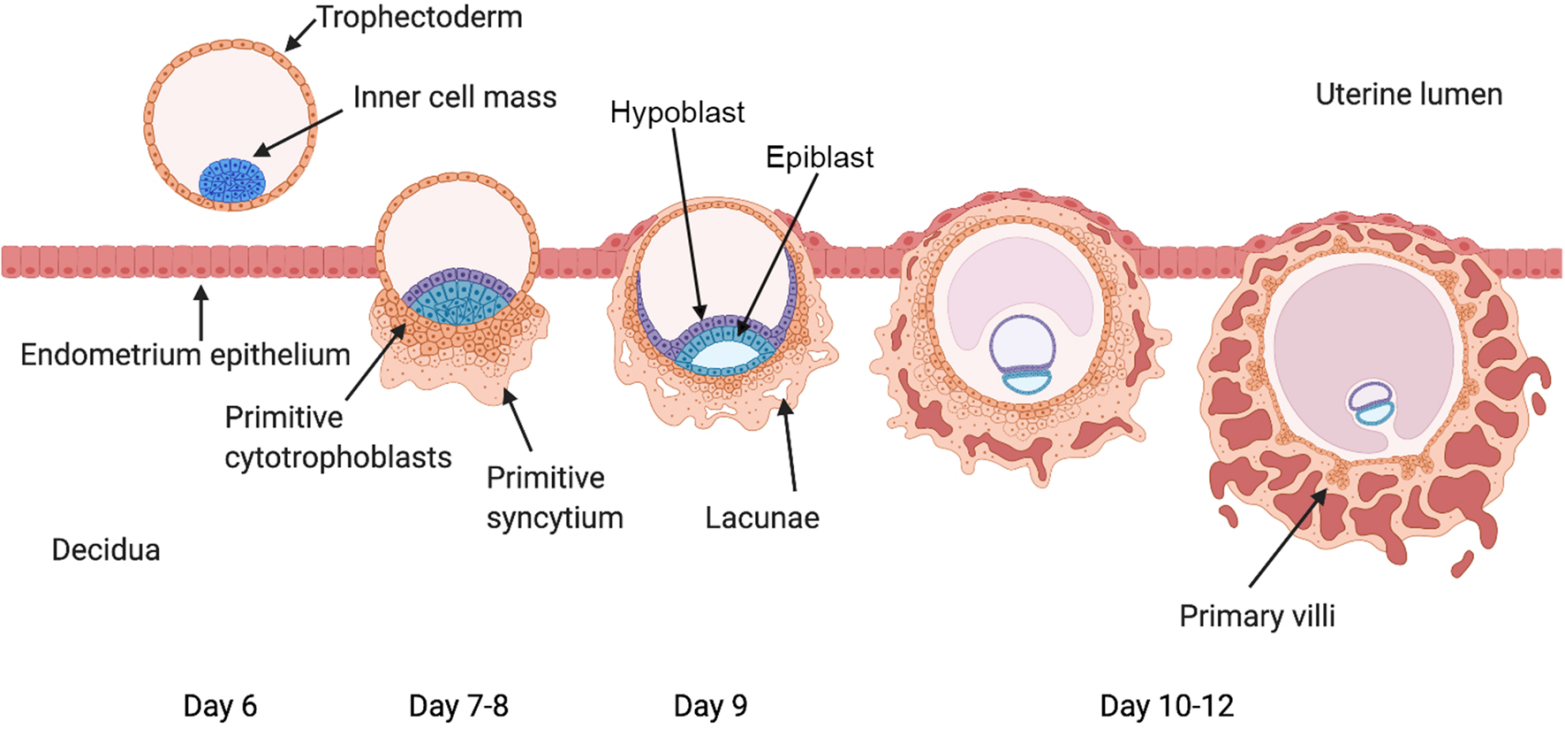
Schematic diagram of blastocyst implantation and early placentation showing development of the first trophoblast lineages. Created with BioRender.com (agreement XV23HRYSE1)

When choosing or developing in vitro models, it is first important to consider what we know of the key cellular events involved in early placentation in vivo. The early developmental events that occur around the time of blastocyst implantation are crucial for establishing the specialised cell types of the placenta. Following fertilisation, the zygote undergoes progressive cell divisions, forming a morula (32 cells), in which the embryonic cells become tightly adhesive and compact, as it travels down the fallopian tube. Simultaneously, the process of decidualization in the second half of the menstrual cycle results in morphological and functional changes that make the decidualized endometrium (decidua) receptive for later implantation [8]. At around 5 days post-conception (dpc), the first embryonic cell lineage differentiation event results in formation of the blastocyst, which is composed of two distinct cell lineages; (1) the inner cell mass (ICM), which forms the embryo proper and is characterised by expression of OCT4 and NANOG, and (2) the outer trophectoderm layer, which forms the trophoblast lineages of the placenta and is characterised by expression of TEAD4, CDX2, GATA2, and GATA3 [9–11]. The polar trophectoderm is a morphologically and phenotypically distinct region of trophectoderm adjacent to the ICM, which at around 6–7 dpc facilitates adhesion of the blastocyst to the endometrial epithelium [6, 9, 12]. Within several hours of attachment, the blastocyst begins to invade into the decidua (Fig. 1). This is initially driven by proliferation and differentiation of the polar trophectoderm, and by 8 dpc two primitive trophoblast lineages are evident; the primitive cytotrophoblast and the primitive syncytium (Fig. 1) [6]. The primitive syncytium is multinucleated and forms an expanding mass that enables invasion of the embryo via the expression of proteases that facilitate extracellular matrix (ECM) remodelling and degradation [12, 13]. By 9 dpc, the primitive syncytium expands to surround almost the entire implanted blastocyst, and the decidual epithelium heals over the top of the implantation site (Fig. 1) [6].

Villus development begins around 12 dpc, with proliferation of primitive cytotrophoblasts, forming expanding projections into the overlying primitive syncytium (Fig. 1) [6]. Around 14 dpc, these projections are invaded by cells from the extraembryonic mesoderm, forming the core of secondary villi [6, 14]. At the same time, primitive cytotrophoblasts continue to expand outwards from the tips of these initial villi, connecting to form the ‘cytotrophoblast shell’ that surrounds the villi, and takes over expansion of the early placenta from the primitive syncytium [6]. Between 15 and 17 dpc, the mesenchymal core develops primitive endothelial tubes, laying the first foundations of the vascular network that will be required for the uptake of oxygen and nutrients, and these villi are then termed tertiary villi [6, 14]. Thus, in contrast to many other organs and tissues (including cancers) in which blood vessels develop first and tissue development follows, in placental development, villus growth precedes vascularisation [15, 16]. By the 4th week of gestation (28 dpc), the basic villous structure of the placenta is evident, consisting of a mesenchymal core containing fetal blood vessels, placental macrophages (Hofbauer cells), pericytes and connective tissue, surrounded by a bilayer of cytotrophoblasts and syncytiotrophoblast [6]. This mature syncytiotrophoblast is quite different to the primitive syncytium and is not invasive, but rather acts as the interface between the maternal circulation and the placenta.

### Trophoblast lineage relationships

All mature trophoblasts in placental villi arise from an upstream trophoblast stem cell (TSC) population. Like other epithelial stem cell populations, it is likely that TSCs themselves proliferate relatively slowly in vivo, but give rise to a highly proliferative ‘transit amplifying population’ (here cytotrophoblasts) that drive placental growth [17, 18]. Cytotrophoblasts exhibit a typical epithelial phenotype, with the majority forming a single layer beneath the syncytiotrophoblast where they adhere to each other via expression of E-cadherin, and to the basement membrane via expression of α6ß4 integrin [17, 19]. Unlike in mice, where TSCs have distinct marker expression patterns from more mature lineages, in the human placenta cytotrophoblasts retain expression of what have been considered key genes for TSC function, including CDX2, ELF5, and TEAD4 [20, 21]. However, isolated cytotrophoblasts can be distinguished by their expression of ß4 integrin, which is not seen on human TSCs [22]. There is evidence that two different sub-populations of cytotrophoblast exist, with those that reside in a monolayer beneath the syncytiotrophoblast primed for differentiation into syncytiotrophoblast, whilst cytotrophoblasts that reside in multilayered clusters in the tips of anchoring villi (characterized by αvß6 or α2 integrin expression) are primed for differentiation into extravillous trophoblasts (EVTs) that migrate out of the placenta and invade into the decidua [23–26]. Finally, it is important to note that whilst the cytotrophoblast: syncytiotrophoblast ratio remains consistent across gestation, significant gene expression and morphological changes are seen in cytotrophoblasts over the course of pregnancy, with cytotrophoblasts flattening against the syncytiotrophoblast and appearing sparse at term [22, 27].

The syncytiotrophoblast is a multinucleated cell layer that covers the entire outside surface of the villous placenta and is in direct contact with maternal blood from late in the first trimester. The syncytiotrophoblast is generated via fusion of underlying monolayer cytotrophoblasts, which exit the cell cycle, and upregulate fusogenic genes such as OVOL1, GCM1, and syncytin-1 [28–30]. In turn, as regions of the syncytiotrophoblast age, they are shed off into the maternal circulation in syncytial nuclear aggregates (SNAs), meaning that like many other epithelial tissues this cell layer is constantly turning over [31]. The primary role of the syncytiotrophoblast is to facilitate exchange between the maternal and fetal circulations, and to achieve this it expresses numerous glucose and amino acid transporters [32]. The syncytiotrophoblast also plays a key role in synthesizing hormones important for maternal adaptation to pregnancy, including hCG, progesterone and human placental lactogen, and further communicates with the maternal system via the release of extracellular vesicles [33]. Finally, the syncytiotrophoblast acts as a barrier between the fetus and its mother preventing free exchange of large molecules and importantly presenting a barrier that is invisible to the maternal immune system due to lack of expression of any HLA molecules.

Cytotrophoblasts at the tips of anchoring villi primed for EVT differentiation (EVT progenitors) undergo a carefully orchestrated programme of differentiation, whereby they progressively gain migratory and invasive capacities [34]. EVT progenitors first differentiate into proximal cell column trophoblasts (pCCTs), identified by expression of NOTCH-1, EGFR and CCR1, that retain their proliferative capacity and adhere to neighbouring pCCTs as they migrate away from the villus in columns [17, 35–37]. Towards the end of the EVT cell columns, distal cell column trophoblasts (dCCT) upregulate Notch2 and activate canonical Wnt signalling pathways via formation of TCF4/β-catenin complexes [17]. This results in dCCT exiting the cell cycle, and undergoing an epithelial-mesenchymal transition whereby they acquire an invasive phenotype via the loss of α6β4 integrin and E-cadherin expression, and gain of expression of α5β1 and α1β1 integrins, and MMP-2 and -9, which together facilitate invasion and extracellular matrix digestion [34, 38, 39]. dCCTs also begin to express HLA-C, HLA-E and HLA-G, which enables interactions between invasive EVTs and maternal immune cells in the decidua to facilitate immunotolerance, an important physiological adaptation during pregnancy [40]. Indeed, HLA-G expression is largely restricted to EVTs, but not expressed by other trophoblasts and frequently used as a distinguishing marker of EVT lineages [17]. Within the decidua, EVT can be further distinguished into distinct subtypes. The two most studied of these are interstitial trophoblasts that are found in the within the decidual stroma, or endoarterial trophoblasts that colonise the uterine spiral arteries and facilitate their remodelling into wide bore conduits that do not respond to maternal vasoconstrictive stimuli. This process of spiral artery remodelling is an important physiological adaptation to reduce the velocity of blood entering the intervillous space and ensure optimal placental perfusion [41–43]. Endoarterial trophoblasts also form loose ‘plugs’ in the spiral arteries during the first trimester, which aid in limiting the flow of oxygenated maternal blood to the intervillous space and create a physiologically normal low oxygen environment favourable for early placental development [43]. In the absence of significant blood flow, histotrophic nutrition derived from glandular secretions provides an important source of nourishment for the developing embryo in early pregnancy [44]. Finally, EVTs are also found in the uterine lymphatic vessels (endolymphatic trophoblast), as well as the uterine veins (endovenous trophoblast) where they are thought to play key roles in eroding these vessels such that they open to the intervillous space to complete this circulation [45].

### Overall considerations in designing in vitro models of trophoblast differentiation or function

A range of models are used to study trophoblast differentiation and function, including explants (ranging from 1–2 mm^3^ to 1 cm^3^ depending on the application) [46–48], enzymatic digestion and/or flow cytometry sorting to isolate specific primary trophoblast populations [49–51], cell lines that can be passaged (including primary TSC lines that exhibit ongoing proliferation [22, 52], and cell lines generated by immortalisation of trophoblasts that otherwise would not proliferate in culture [53, 54]), and choriocarcinoma cell lines [55, 56]. Each of these approaches has application specific considerations. However, regardless of the culture model chosen, there are several broad ranging factors such as cell/tissue sex and viability, cell purity, and the culture medium/microenvironment employed that can influence experimental outcomes. It is thus important to understand the impact these may have when designing experiments.

When using placental explant models, different cells within the tissue may exhibit impaired viability at different points in culture. For many years, the syncytiotrophoblast was thought to rapidly lose viability when exposed to atmospheric oxygen, but within 48–72 h could be regenerated by underlying viable cytotrophoblasts [23, 57, 58]. However, more recent work considering the limitations of traditional viability stains (such as propidum iodide) used in early studies to assess the viability of this enormous multinucleated syncytium demonstrated that this staining is a result of upregulation of pannexin 1 hemichannels, and does not reflect impaired overall syncytiotrophoblast viability [59]. Importantly, alterations in cell viability affect different villous populations in different ways. Production of EVTs from explant models is maintained over at least 5 days of culture, and EVT progenitors remain viable for 2 weeks in culture [23, 46]. In contrast, in the absence of a blood supply, viability of the mesenchymal core of villi is limited to 48 h, impacting an important source of paracrine signalling [23]. Overall, the above considerations highlight the importance of individual researchers characterising their models, and understanding how viability may impact the endpoints being assessed in the specific conditions employed in their work.

Whilst explants recapitulate the 3D environment and retain important cell relationships within the placenta, isolating and culturing specific trophoblast populations is also important to describe trophoblast function/dysfunction, in particular as gene/protein data acquired from explant lysates contains a high proportion of mesenchymal and vascular cells that may significantly confound results [60]. When isolating primary trophoblasts, it is important to determine the purity achieved by the method employed, and in particular that contamination with mesenchymal cells (which grow more easily and rapidly in a range of conditions) is avoided. This can be achieved at the most basic level by double labelling for cytokeratin-7 (an exclusive marker of trophoblast in the placenta) and vimentin (expressed by mesenchymal and vascular cells). Prevention and/or characterisation of mesenchymal contamination is particularly important in EVT cultures, which acquire a more migratory mesenchymal morphology as they differentiate, and here HLA-G is useful to clearly distinguish EVT lineages. Isolation of trophoblast by FACS enables cells to be selected based on lineage markers (HLA-G for EVTs, β4 integrin for cytotrophoblasts) thereby reducing contamination [51], but does have drawbacks in terms of cost and impact on cell viability.

Beyond basic considerations of cell viability and expression of key lineage markers, the in vitro culture conditions employed have the potential to impact cell health and function. This is particularly evident in the study of trophoblast mitochondrial activity, where exposure to non-physiological oxygen conditions, altered glucose levels, or culture media deficient in micronutrients, such as selenium, that aid mitochondrial function may negatively impact overall cell metabolism [61–64]. Considerations of oxygen tension in relation to in vitro models of placentation are particularly important, as the limited blood flow to the intervillous space for most of the first trimester means that the placenta exists in physiologically normal low oxygen conditions (18 mmHg, or ~ 2% oxygen) [65]. In contrast, after the intervillous circulation is fully established at 13–14 weeks, the placenta is exposed to ~ 8% oxygen (60 mmHg), slightly lower than the PO_2_ of the uterine artery feeding towards the intervillous space (~ 90 mmHg) [65–67]. It is important to remember that the placenta both takes up and consumes oxygen, and thus PO_2_ measurements within the intervillous space decrease as the placenta grows, reaching ~ 30–40 mmHg near term [67]. Furthermore, oxygen levels can vary in pregnancy disorders such as FGR where oxygen delivery and placental oxygen saturation is consistently lower, or pre-eclampsia where oxidative stress results from fluctuating oxygen levels creating a hypoxia-reperfusion scenario [68]. In reality, oxygen levels are also heterogenous within the intervillous space, making oxygen tension difficult to ever model perfectly in vitro [67]. However, it is important in the least to consider the context of what is being modelled when selecting which oxygen conditions to employ or compare.

Culture media composition can also have important impacts on cell phenotype and function (including marker expression, proliferation, and differentiation), although while this has been assessed in the context of placental mesenchymal stromal cells [69], and specific conditions have been defined to induce extravillous trophoblast or syncytiotrophoblast differentiation [70], the impact of different basal media on trophoblast phenotype has not been studied in detail. Nonetheless, as single cell technologies allow distinct trophoblast sub-populations to be resolved that may have unique trophic niches and functions in vivo [71], understanding the impact of culture media and accurately recapitulating distinct microenvironments in vitro may be important to accurately study lower abundance trophoblast populations in the placenta, and/or prevent phenotypic convergence in culture [72]. Finally, understanding the impact of culture media on both primary trophoblasts and trophoblast cell lines will become increasingly important as more anatomically accurate but complex co-culture models are developed that employ multiple cell types (endometrial stromal or epithelial cells, vascular cells, immune cells), but for which a single culture medium may be required.

It is becoming increasingly apparent that sex-specific differences in placental/trophoblast function exist that may affect experimental outcomes. Such effects may in part arise as a result of differential gene methylation [73], and span a range of cellular processes, including mitochondrial function, metabolism, cytokine expression, steroid hormone sensitivity, and growth signalling pathways [74–76]. Indeed, sex-specific differences in placental function are emerging as an important factor impacting fetal health, and may play a role in the development of pregnancy disorders [77, 78]. Thus, regardless of the model and conditions chosen, where possible replicates should be sufficient to do independent analyses by fetal sex to determine if such an effect exists. Whilst with term placentae the fetal sex can be immediately known, for first trimester placentae sexing of the tissue may need to be done retrospectively via PCR analysis of the SRY gene. At the cellular scale, sex-specific differences have been reported from the stem cell level, and thus have the potential to impact all downstream trophoblast lineages [79]. Thus, sex-specific factors should also be kept in mind when using cell lines established from primary tissues (e.g. isolation of TSCs), or using or generating new immortalised trophoblast lines.

Finally, whilst the focus of this review is on primary trophoblast models, in the absence of access to primary tissue, many laboratories rely on cell lines to model trophoblast behaviour and understanding of the similarities and differences between cell lines and primary trophoblast is thus important to accurately interpret data. It is important to note that trophoblast cell lines can vary in a range of ways both from other cell lines, and from the primary cell they endeavour to model [80–82]. For example, choriocarcinoma cells (Jar, Jeg3, BeWo) exhibit extensive chromosomal abnormalities, and their extended propagation (over 50 years) has led to phenotypic drift, resulting in reports of differences in maker expression (e.g. HLA-G) between locations and sources. Where appropriate, differences of importance to modelling of individual trophoblast lineages, and variations from primary models that may affect data interpretation, will be highlighted in the relevant section below.

### Models of TSC

Early stem cell models of human trophoblast differentiation were dependent on embryonic stem cells (hESC), that could be induced to differentiate into mixed populations of syncytiotrophoblast and extravillous trophoblast via treatment with BMP4 (reviewed in [10]). Such models have a number of limitations but have been improved in recent years by approaches employing naive pluripotent/embryonic stem cells, which have not yet committed to a trophectoderm of ICM lineage [83–85]. Such models have enabled the generation of human TSCs similar to those derived from blastocysts and first-trimester placentae. However, whilst improvements in iPS-derived trophoblast models provide important insights into the generation of trophectoderm and the molecular pathways regulating TSC formation [85], and may help enable more accurate modelling of placental pathologies [86], it is equally important to pursue our understanding of primary human TSCs in vivo and in vitro to understand the ground truth being modelled by these other systems, and this will be the focus of this review.

Like many other stem cell populations in humans, our understanding of the potential role of human TSCs in the placenta began in murine studies, from which TSCs were first isolated and cultured in 1998 using cells derived from mouse extraembryonic ectoderm (ExE), a tissue of trophectodermal origin that gives rise to the mouse trophoblast lineages [87]. Here, the culture of murine ExE cells in the presence of fibroblast growth factor 4 (Fgf4) resulted in the emergence of a highly proliferative epithelial colonies that expressed Errb, Cdx2, Fgfr2, and eomesodermin (Eomes) [87]. Differentiation to mature murine trophoblast lineages could be induced by removing Fgf4 or adding heparin, resulting in a decline in proliferation and a change in morphology to a giant-cell phenotype (a murine-specific trophoblast lineage) [87]. In experiments using chimeric mouse embryos, TSCs contributed only to aspects of the trophoblast lineage, and were not observed in the ICM or other germ layer derivatives, confirming their commitment to the trophoblast lineage in early differentiation [87]. Establishment of a murine TSC model enabled researchers to understand key gene networks that maintain murine TSCs in an undifferentiated state (Oct4, Ets2, Fgf4, Elf5), or are key regulators of trophoblast differentiation (Tfap2c, Eomes, Cdx2, Gata3) [21, 88].

Despite the above advances in murine TSC, differences in the structure, early development, and gene/protein expression between the mouse and human placenta mean that caution must be used in directly translating these findings to the development of human trophoblast lineages. Indeed, human TSCs could not be successfully isolated/propagated from blastocysts using the conditions employed for murine TSC [89], and the first successful generation of a human TSC line came from the isolation of blastomeres from morula stage embryos almost 2 decades after murine TSC were propagated [90]. This UCSFB6 cell line spontaneously generated cells that expressed transcription factors characteristic of human trophectoderm and/or TSC (cytokeratin 7, TEAD4, CDX2, GATA3, ELF5), although expression of EOMES, a marker absent from human trophectoderm/trophoblast lineages, was also reported [20, 90]. When cultured on Matrigel, some cells formed multinuclear syncytiotrophoblast-like aggregates, while mononuclear migratory cells invaded into the Matrigel and expressed EVT markers (HLA-G, α1 integrin) [90]. However, the inability of many other labs to obtain morula stage human embryos and screen the resulting range of cell lines means the methodology to isolate similar cell lines was not widely adopted.

#### Isolation of TSCs and trophoblast progenitor populations from human placenta

TSCs are also present within the early human placenta, providing opportunities to isolate them from this tissue. In humans, the first postulated trophoblast progenitor cell (TBPC) line was derived from first trimester chorion in 2011 [91]. TBPCs formed colonies with an epithelial morphology, were more invasive and proliferative than primary cytotrophoblasts, and expressed cytokeratin 7, α4 integrin, OCT4 and GATA4, but did not express the TSC marker CDX2 [91]. When differentiated on Matrigel using growth medium supplemented with FGF4 and EGF, some TBPCs aggregated and fused into multinucleated structures, that expressed syncytiotrophoblast markers (syncytin, GCM1, hCG) [91]. However, the majority became migratory and expressed EVT markers (HLA-G, α1 and α5 integrins), with these resulting cells postulated to represent the migratory trophoblast populations present on the chorion [91, 92]. In 2015, a further novel population of trophoblasts was isolated from first trimester placental villi using the Hoechst side-population technique, which has previously been used to isolate stem cells and progenitors from a variety of tissues [51, 93, 94]. Side-population trophoblasts sorted directly from tissue by flow cytometry form a transcriptomically and methalomically distinct population that comprised around 3% of mononuclear villous trophoblasts, were more closely related to cytotrophoblast than extravillous trophoblast, and expressed genes indicative of a TSC population (TEAD4, ELF5, CDX2) [51, 95].

In 2018, a significant breakthrough in this field was made when Okae et al. isolated and propagated the first widely accepted ‘true’ TSC from both human blastocysts and first trimester trophoblast digests [52]. The culture medium used in this work (allowing the activation of Wnt and epidermal growth factor (EGF) signalling, while simultaneously inhibiting Rho-associated protein kinases (ROCK), histone deacetylase and TGF-β activity) was key to this achievement by enabling proliferation and prolonged culture of cytotrophoblasts, which in turn promoted the subsequent expansion of TSCs within these cultures, allowing their purification [52]. Both, blastocyst and first trimester derived TSCs expressed markers of trophoblast identity (including cytokeratin 7, TP63, TEAD4, GATA3), had lower overall gene methylation than cytotrophoblasts (as is a feature of many stem cell populations), and like murine TSCs, exhibited hypomethylation of the ELF5 promoter [17, 52, 96]. The TSC isolated by Okae et al. have a robust proliferative capacity, and can be maintained undifferentiated in culture long term, or differentiated into hCG+ multinucleated syncytiotrophoblast (via exposure to medium supplemented with forskolin, a cAMP agonist), or HLA-G + migratory EVTs (via exposure to media lacking the Wnt activator CHIR99021), with both TSC-derived mature trophoblast populations exhibiting transcriptomic similarities to primary EVTs and syncytiotrophoblast [52]. In vivo, injection of the TSCs into 6–8-week-old non-obese diabetic mice resulted in lesions that were found to contain α6 integrin+ cytotrophoblast-like cells, migrating EVT-like mononuclear cells, and SDC1+ syncytiotrophoblast-like cells containing lacunae (the gaps between arms of the primitive syncytium during implantation) [52], all key features of early placentation in vivo. This ability to initiate controlled, lineage-specific differentiation of trophoblasts provides significant advantages over prior hESC models of trophoblast lineage differentiation, and the lack of specialist equipment required to derive Okae TSC, and/or their commercial availability for laboratories that cannot access first trimester tissue, means that the cells and culture conditions employed by Okae et al. are increasingly becoming the preferred model for in vitro studies of trophoblast differentiation by many researchers.

The work of Okae et al. has also aided the development of other human TSC models. Whilst side-population trophoblasts were unable to be successfully cultured in complete trophoblast medium or multiple human pluripotent stem cell media, the TSC medium employed by Okae et al. [52] enabled first trimester side-population trophoblasts to be propagated and passaged, demonstrating that whilst initial growth is relatively slow, their growth kinetics increase with time in culture [22]. This suggests that the rapid proliferation of Okae TSCs may be a culture response, rather than an in vivo feature of TSCs. Furthermore, cultured side-population trophoblasts exhibited the differentiation potential of a TSC population, and could be directed to differentiate via β4 integrin + cytotrophoblasts, into multinucleated hCG+ and syncytin-1+ syncytiotrophoblast, or HLA-G+ EVTs that invade through Matrigel [22]. A key difference between the derivation of TSC using the side-population technique compared to the Okae protocol is that the side-population technique allows direct isolation from placental tissue, without extended culture and adaptation to in vitro conditions that is required in the Okae et al. protocol [22, 51]. Thus, this work may provide complimentary insight to the in vivo phenotype of TSCs.

#### Organoid models

The presence of TSCs in first-trimester placenta has enabled the development of 3D trophoblast organoid models capable of self-renewal, expansion and differentiation [97, 98]. These organoids are originally derived from isolated mononuclear villous trophoblasts (predominantly cytotrophoblasts, but containing a subset of TSCs), and it is thought that culture conditions either support cytotrophoblast proliferation and the ongoing differentiation of TSCs feeding into this pathway to drive organoid expansion, or promote the growth and development of TSCs within the starting cultures, such that they become the dominant proliferative cell type capable of differentiating into mature lineages [99]. Unpicking the relationships between cytotrophoblasts and TSCs within placental organoids is confounded by the fact that in humans there is extensive overlap in markers expressed by both these cell types, and further analyses employing markers that distinguish TSCs and cytotrophoblasts (such as β4 integrin) is required to truly understand the relative proportion of these populations within organoids. In comparison to a placental villus, trophoblast organoids have an inverted architecture, with p63 expressing cytotrophoblasts on the periphery, which undergo spontaneous fusion to form ENDOU, GCM1 and hCG-positive multinucleated syncytiotrophoblast in the centre of the organoid [97, 98]. In line with this, there is a progressive reduction in cell proliferation from the outer edge to the centre of the organoid, which is believed to be associated with fusion and terminal syncytiotrophoblast differentiation [97]. Removal of Wnt signalling, which maintains epithelial proliferation and stemness, induced development of Notch1+ HLA-G+ EVTs that grow out from the organoid and invade through the culture matrix [97]. Alternatively, elevating the activin/nodal pathway inhibitor A83-01 in the absence of valproic acid and EGF was also able to induce EVT production [98]. Despite their inverse architecture, 3D organoid models provide important advantages over two-dimensional (2D) cell culture by allowing specific cell–cell interactions and the mechanical cues that result from structural complexity during early placentation to be modelled [100].

The reduced ability to generate trophoblast organoids from placentae > 10 weeks of gestation [97, 98], combined with the inability of Okae et al. to derive TSCs from second or third trimester placentae using conditions employed for first trimester TSCs, led to the hypothesis that TSCs do not persist throughout gestation [52, 97, 98]. However, TSC-like cells have been isolated from mid-gestation murine placentae [101], and an alternate possibility is that second or third trimester TSCs have different trophic requirements in culture to those from early gestation tissues. Indeed, placental villi and cytotrophoblasts undergo significant changes in morphology across gestation, and the transcriptome of cytotrophoblasts differs between the first and third trimesters [6, 22]. However, this barrier of culture conditions can be circumvented by use of the side-population technique, which can isolate cells directly from placental tissue, and indeed side-population trophoblasts can be isolated from term placentae, where they constitute a similar proportion of viable mononuclear trophoblasts as they do in the first trimester [22]. Furthermore, side-population trophoblasts were reduced tenfold in FGR placentae [22]. Further understanding of the distinct niche of late gestation TSCs will aid the development of culture conditions to propagate these cells. Recently, retroviral constructs have also been used to induce/increase expression of GATA3, TFAP2C, TEAD4, CDX2, ELF5, ETS2 in term cytotrophoblasts, generating ‘iTSC’ lines that can differentiate into extravillous trophoblast or syncytiotrophoblast [102]. Whilst cytotrophoblasts already exhibit basal expression of these markers, here it appears that inducing higher levels of expression results in iTSC lines that, unlike term cytotrophoblasts, can be passaged in TSC media for extended periods [102]. Evidence of the persistence of TSCs across gestation, and continued development of models to generate TSC from term placentae, will allow for the development of improved disease models of TSC dysfunction in pregnancy pathologies.

### Models of syncytiotrophoblast

In humans, the syncytiotrophoblast is an important trophoblast subtype. It is in direct contact with the maternal blood, thus acting as an interface for fetal-maternal interaction such as nutrient and gas exchange. Therefore, creating a syncytiotrophoblast model is necessary to help understand human placental function; however, it is currently challenging as the syncytiotrophoblast is a large, multinucleated, single cell covering an area of approximately 11–13 m^2^ [103]. Additionally, the syncytiotrophoblast is unable to proliferate and instead relies on the fusion of the underlying cytotrophoblast for regeneration and expansion, and thus any damage in the isolation process will compromise the integrity of the syncytiotrophoblast [103]. Early work in the field recognized the difficulty of directly isolating the intact layer of syncytiotrophoblast, and instead focused on refining the isolation method via a combination of mechanical dissociation, enzyme digestion, and serial agitation, with only partial success [104, 105]. Trypsin was an attractive digestion enzyme as it can release and preserve the integrity of most placental cells, but it was noted to be harmful and can destroy the syncytiotrophoblast [104]. An alternative approach is to use a solution of 0.03% EDTA, which was shown to detach the syncytium adequately, although results can vary between placentae [105].

**Fig. 2:**
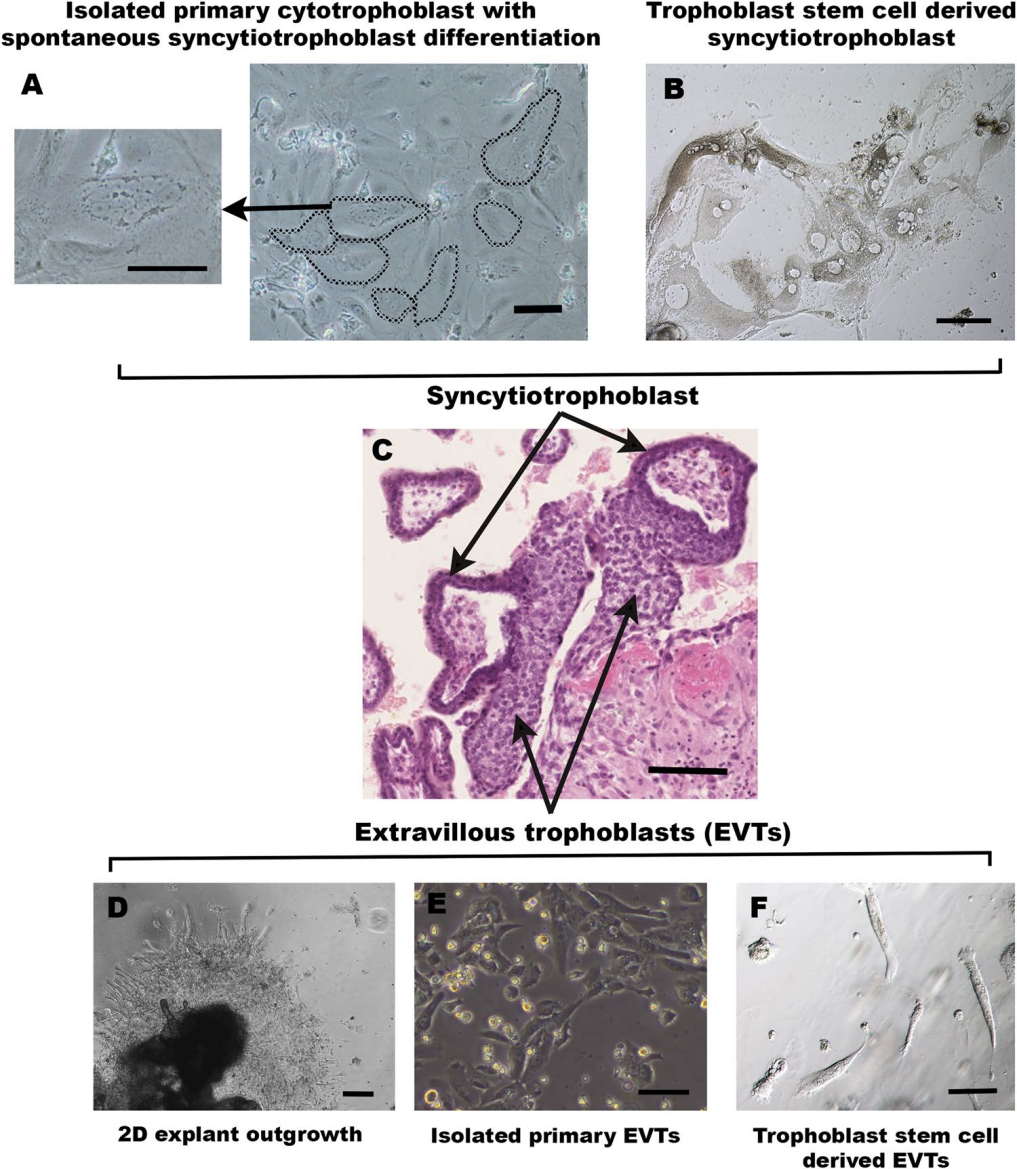
Examples of in vitro culture models of syncytiotrophoblast and extravillous trophoblast (EVT). In vivo anatomy of the syncytiotrophoblast and EVT from a 7.6 week of gestation placenta is demonstrated in **C** (image captured from the Boyd Collection, Centre for Trophoblast Research, University of Cambridge). Cells labelled as EVTs indicate EVT lineages in the cell columns (arrowheads point to dCCT). Invasive interstitial EVTs will also be present in the decidua but are difficult to accurately distinguish in this H&E image, and thus are not separately labelled. **A** Phase contrast images of cytotrophoblast from a placenta of 39 weeks of gestation isolated using the protocols of [110], in which spontaneous syncytiotrophoblast differentiation has occurred over the 30-day culture period. Multinuclear regions of syncytiotrophoblast are circled, with one example shown at a higher magnification. **B** Hoffman modulation image of syncytiotrophoblast differentiated from TSC isolated from an 8.5-week placenta using the side-population technique, as described in [22]. **D** Phase contrast image of EVT outgrowth from villous explant from a 9.1-week placenta extending across a thin layer of Matrigel (model as described in [46]). **E** Phase contrast image of primary EVTs isolated from a placenta of 8.2 weeks of gestation cultured on a thin layer of Matrigel using the technique described in [156]. **F** Hoffman modulation image of EVT differentiated from TSC isolated from an 8.4 week placenta using the side-population technique, as described in [22]. Scale bars on all images represent 100 μm

**Fig. 3:**
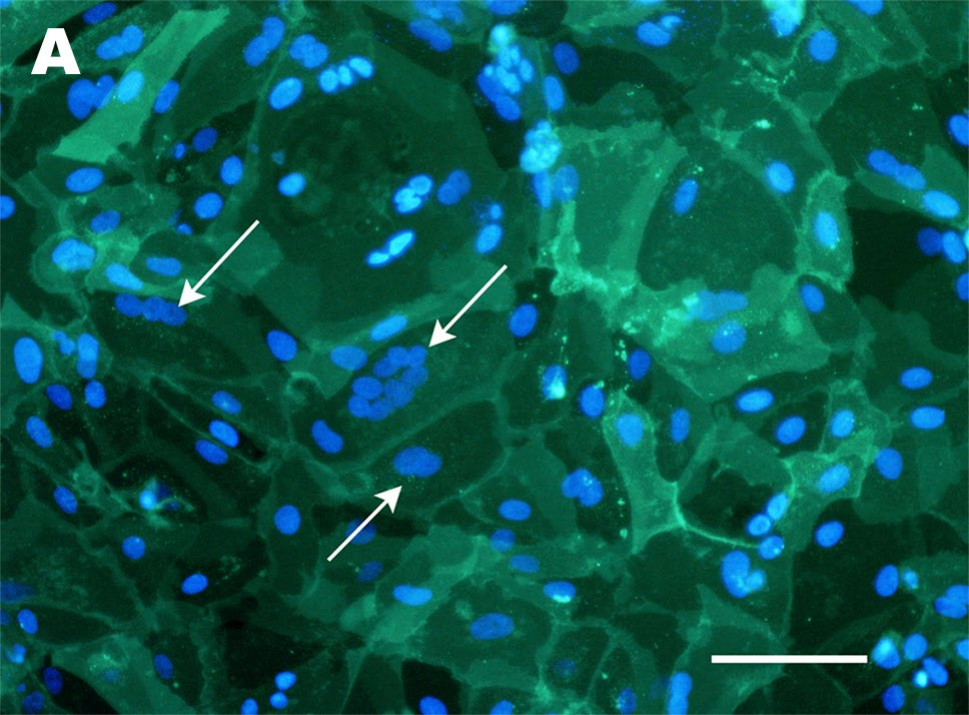
Confirmation of syncytialisation by staining of cell membranes. **A** Fluorescent image of PKH67 staining of cultures of cytotrophoblasts from a placentae of 39 weeks of gestation (isolated using the protocols described in [110]), in which spontaneous syncytiotrophoblast differentiation has occurred over the 30 day culture period. Nuclei are counterstained with Hoechst 33342. Multinuclear clusters within a single membrane (demonstrating syncytiotrophoblast) are indicated with white arrows. Scale bar = 100 μm

The above issues with direct syncytiotrophoblast isolation can be circumvented by culturing cytotrophoblasts, which are capable of spontaneous fusion into multinucleated syncytiotrophoblast-like clusters in vitro [106, 107]. The method of growing naturally fused trophoblast is an important development in trophoblast culture. However, a significant downside to the technique is that freshly isolated cytotrophoblasts lose their proliferative capacity when cultured in vitro [107]. Upon isolation, other proliferative, non-trophoblast contaminants, such as fibroblasts, can overtake the culture within 7–10 days [107, 108]. Methods to reduce this contamination include Percoll gradient separation [50] and negative selection by antibody-coated magnetic beads [109]. Still, these additional steps do not extend the cytotrophoblast culture period beyond seven days. Recent findings showed that it is possible to culture human trophoblast for up to 30 days by manipulating the seeding density and duration [110]. This method allows the generation of multinucleated syncytialised clusters and enabled maintenance of the cultures in vitro for at least 30 days without becoming overgrown by contaminating cells [110]. However, prolonged survival of trophoblasts in culture revealed that even after 30 days, not all cytotrophoblasts will naturally fuse, and up to 50% will remain mononuclear (Fig. 2) [110]. This highlights a key point to remember when working with syncytialised trophoblasts in culture—the need to demonstrate that cell clusters are truly syncytialised, and are not just colonies of tightly packed mononuclear trophoblast. To confirm syncytialisation, it is essential to stain the putative syncytial clusters to demonstrate the absence of cell membranes between the nuclei. This has often been achieved by immunohistochemistry employing antibodies that recognise cell membrane proteins such as E-cadherin or desmoplakin [22, 111, 112]. Alternatively, more rapid and sensitive lipid stains, such as PKH67, can also be used (Fig. 3) [110].

Whilst standard, static, 2D primary culture models have provided valuable tools to study the syncytiotrophoblast, the development of culture systems that more closely recapitulate physiological conditions is key to further understand syncytiotrophoblast function. Whilst difficult to mimic all the complexities of the in vivo placenta simultaneously, cultures have been designed to incorporate elements of placentation such as fluid shear stress, transport across the syncytiotrophoblast barrier, and/or cell–cell relationships. The flow of maternal blood flow through the intervillous space means that in vivo the syncytiotrophoblast is constantly exposed to fluid shear stress, which in other cell types (e.g. endothelial cells) has important impacts on cell function [113]. Indeed, primary human syncytiotrophoblasts express mechanosensitive channels and proteins, and exposure of syncytialised BeWo to shear stress in microfluidic culture systems results in the upregulation of microvilli on their surface [114, 115], although wider downstream consequences of shear stress on syncytiotrophoblast function are largely unknown. The impact of shear stress on the syncytiotrophoblast has also been studied using bespoke systems in which villous explants were secured to the bottom of flow chambers, demonstrating that fluid flow helped maintain ultrastructural integrity of the tissue for longer than static conditions [116]. Finally, rotating wall bioreactors have also emerged that both simulate fluid flow and recreate the suspended, 3D, microgravity environment found within in vivo tissues [117]. A key consideration in designing microfluidic or larger scale flow cultures of syncytiotrophoblast is the need to consider physiological ranges of shear stress the tissue is exposed to at the relevant point in gestation (predicted by computational models such as in [113]), and to carefully design systems that recapitulate this as accurately as possible, something that becomes more mathematically and practically challenging as shear stress systems are paired with more anatomically accurate 3D cellular structures.

‘Placenta-on-a-chip’ models of syncytiotrophoblast transport function that combine multiple cell types and culture compartments are also a key area of interest. In these scenarios, microfluidic channels with integrated electrical sensors allow the integrity of cell monolayers to be evaluated, and enable measurement of the exchange of continuous flowing nanoparticles across trophoblast cell lines [118, 119]. Dual 2D layers of endothelial cells and trophoblast (to date represented using syncytialised BeWo) are seeded either side of a semipermeable membrane, recapitulating the transport barrier from the maternal to fetal circulations at sites where the capillaries protrude to the villous edge. Fluid flow through separate microfluidic channels either side of this cell barrier enables both exposure to cell specific medium, and quantification of transport across the layer [120–122]. Finally, a key component of syncytiotrophoblast function involves cell turnover—with cytotrophoblasts fusing in to the syncytiotrophoblast, and parts of the syncytiotrophoblast shedding off into the maternal circulation. Studying this aspect accurately requires 3D models that recapitulate this trophoblast bilayer. However, existing trophoblast organoid models (discussed earlier) are limited in this application, as their inverted architecture means the syncytiotrophoblast does not have an outer apical membrane exposed to culture medium, and thus does not shed in the normal manner. Thus, there remains a need to continue to develop alternate 3D models that more accurately mimic the trophoblast bilayer that may better enable syncytiotrophoblast turnover to be studied.

To date, most studies employing more complex microfluidic or 3D culture models have used trophoblast cell lines, likely due to their ongoing proliferative capacity and reduced requirements for culture on specific extracellular matrices, which makes them easier to seed into more complex culture systems. The application of primary trophoblast to the next generation of culture systems profiled above would aid their physiological accuracy. Some success in creating functional physical barriers from primary cytotrophoblasts has been achieved by undertaking multiple seeding cycles of revived cytotrophoblasts into established trophoblast cultures at different intervals, creating overlapping layers of trophoblast at varying stages of differentiation and imitating the continuous cellular life-cycle of cyto/syncytiotrophoblast [123]. However, this causes cells to aggregate, leaving behind large intercellular areas and preventing formation of a monolayer connected by tight-junctions [123]. Such aggregates could also cause blockages in microfluidic systems. The application of proliferative TSCs to such models in the future may help overcome these issues by enabling proliferation within microfluidic systems prior to differentiation, although this is not without its own technical challenges as syncytiotrophoblast differentiation protocols can take up to 9 days.

### Models of EVT

The role of inadequate EVT migration, invasion and spiral artery remodelling in the pathophysiology of pre-eclampsia and FGR, and of excessive EVT invasion in placenta accreta spectrum, has led to a range of in vitro modelling approaches to better understand how EVT differentiation and invasion is regulated, and why this may differ in pathology. Here, it is important to note that three distinct processes can be assessed; (1) the generation of EVTs from their progenitors, (2) the migration of EVTs across a 2D surface, or (3) the invasion of EVTs through 3D extracellular matrix. Each of these processes is regulated or facilitated by different (although at times overlapping) molecules, and as such it is important to be aware of what the model being employed is assessing when interpreting results.

First trimester explants have been used to generate EVT outgrowths in vitro using several different systems [46, 48, 124]. Typically, villous tissue is washed and dissected into relatively small explants (several mm^3^). Some researchers use a dissecting microscope to select for villi with existing EVT outgrowths to maximise potential yields [124], whilst others randomly select much higher numbers of explants, enabling rates of production of EVT outgrowth (i.e. the overall placental capacity for EVT generation) to be quantified [46, 125]. Some researchers embed explants within Matrigel domes, meaning that EVT outgrowths form in 3D as they do in vivo (potentially recapitulating the development and cellular organisation of EVT columns more precisely), and must invade through the extracellular matrix to expand (thus accurately reflecting invasive capacity) [124, 126, 127]. However, accurate quantification of such outgrowths in 3D is more challenging, with gross 2D measurements of outgrowth diameter or length usually provided as a proxy measure [124, 126, 127]. Other researchers plate explants on a thin layer of Matrigel, meaning that outgrowths expand in 2D across the surface, providing a measure of EVT migration rather than invasion (Fig. 2) [46, 125]. Whilst less physiologically accurate than 3D models, this does enable accurate high-throughput quantification of outgrowth area across larger numbers of villi, mitigating against biological variability [46, 125]. Finally, following culture, fixation and sectioning of intact explants and outgrowth allows more detailed immunohistochemical investigation of the EVT progenitors within the tips of villi, allowing researchers to correlate the extent of outgrowth with the molecular phenotype of upstream progenitors [35].

As EVTs leave the proximal cell columns and move away from the villus they exit the cell cycle and lose the ability to proliferate, both in vivo*,* and in in vitro explant models [38, 124, 128]. Thus, the expansion of EVT outgrowths from villous explants is driven by differentiation of progenitors into EVTs, and subsequent proliferation of pCCT, rather than by proliferation of the majority of EVTs within the outgrowth. This has at times been disputed by the observation that EVT outgrowths in explant culture stain with proliferating cell nuclear antigen (PCNA) [129]. However, the 20 h half-life of PCNA means it can be detected in cells that stopped replicating 24–48 h earlier [130], and thus outgrowths generated by upstream pCCT proliferation in the first 1–2 days of short-term explant culture retain PCNA expression. A more accurate reflection of actively proliferating cells is provided by staining for Ki67, which is only observed in cells close to the villus [128].

The fact that terminally differentiated EVTs do not proliferate has important overall implications for the use of primary EVTs in in vitro models, as it limits the yield and lifespan of isolated cells, meaning that frequent labour-intensive primary isolations are required. Whilst some authors have reported that isolated cytotrophoblasts plated on Matrigel can differentiate into EVTs [131], isolated cytotrophoblast are generally considered to preferentially and spontaneously syncytialise in culture [132, 133]. As such, direct enzymatic isolation of EVTs from first trimester placentae has been more frequently adopted to generate sufficient cell yields for downstream applications (Fig. 2) [49, 125]. However, due to the size of first trimester samples, yields are often limited (typically < 400,000 cells) and EVTs isolated by common methods do not proliferate when plated [125, 133]. It is also important to note that EVTs isolated from first trimester placental villous tissue arise from the EVT cell columns, and are not yet actively invading within decidual tissue. Thus, whilst their differentiation programme likely continues in vitro via contact with an appropriate extracellular matrix (e.g. Matrigel, collagen), direct transcriptomic analyses may be more reflective of proximal or distal CCT. Indeed, isolation of proliferative pCCT from first trimester placentae that are both Ki67+ and HLA-G+ has been reported [134, 135], however as in vivo HLA-G is upregulated in dCCT that are exiting the cell cycle [37], further phenotypic characterisation of these cells is required. Isolation of EVTs is also possible from term placentae via enzymatic digestion of the basal plate, although isolated term EVTs only remain viable in culture for around 48 h, making them more suitable to immediate gene/protein analyses than in vitro culture [136, 137]. Recent developments in the TSC field have made significant impact in overcoming issues regarding the limited lifespan of isolated primary EVTs across gestation, or access to primary tissue, by enabling TSC to be propagated and then differentiated to HLA-G+ EVTs (Fig. 2) [52], and this is increasingly being adopted as a more physiologically accurate alternative to transformed (HTR8/SVneo, SGHPL4) or choriocarcinoma (Jar, Jeg3) cell line models of EVT.

EVTs can be employed in a number of in vitro assays designed to assess their migratory or invasive function. The simplest of these is in wound healing assays, a high-throughput assay adopted from the cancer field, where a scratch is made in a 2D cell-line monolayer and the migratory capacity of cells is quantified by the rate at which they fill this space, or the proportion of space occupied by cells at a fixed timepoint [138, 139]. However, caution is warranted when proliferative cell lines are used in this setting, as the effects of cell proliferation vs cell migration cannot be separated. Seeding explants or EVTs on the top of Boyden chambers (Matrigel-coated polycarbonate membrane Transwell inserts (usually 8 or 12 μm pore size)) enables quantification of the number of cells that invade through the Matrigel to the lower chamber over 24–72 h of culture, and provides a more robust assessment of true invasion [140, 141]. Whilst these traditional models consider migration across or invasion through an extracellular matrix, the success of EVT invasion arises as a result of a complex network of signalling between the decidua and the invading EVTs [142]. In light of this, researchers have looked to further improve the physiological accuracy of EVT invasion models by incorporating multiple cell types. In basic systems, trophoblasts—either in single cell suspensions or in spheroids—are seeded on top of endothelial or stromal cell monolayers [143–145], but it is also possible to co-culture trophoblasts with segments of uterine spiral arteries [146], or villous explants with decidual tissue to examine cellular interactions [147, 148].

Interactions of EVTs with decidual immune cells are also physiologically relevant, and occur by direct cell–cell interactions and by paracrine mechanisms [149]. Immune populations such as decidual natural killer cells or macrophages can be isolated directly from first trimester decidual tissue and co-cultured with isolated EVTs [150, 151]. Whilst co-culturing of matched isolates of primary cells is the gold standard for this work, many researchers employ EVT cell lines in such models, perhaps due to the separate time-intensive cell isolation protocols required for each cell type, or to standardise one aspect of the system. Separation of the effects of direct cell–cell interactions from those induced by paracrine mechanisms can also be undertaken by culturing EVTs (isolated or in explant outgrowths) with media conditioned by immune populations [152, 153]. Finally, some authors have examined the spatial relationship between invading EVTs and decidual immune populations by co-culturing first trimester placental villous tissue with intact pieces of decidua peritalis [154], and such models that enable cellular relationships within the decidua to be maintained could be extended to other analyses.

Microfluidic models provide further advantages in assessing trophoblast migration/invasion in complex environments by enabling precise control of chemotactic gradients and tracking of cells in real time, resulting in the generation of rich stochastic datasets to describe cell invasion/migration (speed, directionality) and cell–cell interactions. Importantly, the small volumes and numbers of cells required for microfluidic systems makes them amenable to the use of primary EVTs [155, 156]. The design of such models depends on the biological question being asked, but important considerations include the cell types employed, stiffness and composition of the 3D matrix components used in relation to the tissue being modelled, and media composition that enables viability and/or proliferation of multiple cell types. At the simplest level, migration of EVTs along endothelial cell monolayers in microfluidic channels has been used to consider how colonisation of the spiral arteries occurs, and quantify the influence of shear stress on trophoblast migration and trophoblast-induced endothelial cell apoptosis during this process via time-lapse microscopy [156, 157]. When considering invasion of EVTs through the decidual stroma, cells may be embedded in physiologically relevant hydrogels, with different culture medium circulating through channels on either side, enabling cell invasion towards chemotactic factors (e.g. GM-CSF) to be quantified in 3D [155]. More complex organ-on-a-chip models are also being developed to study trophoblast invasion that incorporate multiple cell types (including stromal cells, endothelial cells and/or uterine natural killer cells) to assess their respective impacts on EVT phenotype and invasion [134, 158]. In comparison to tissue co-culture models, these multicellular microfluidic models are more accessible to direct imaging and quantification of EVT invasion, and enable different experimental setups that can layer in the effects of different combinations of decidual and immune cell populations to more fully understand the contribution of each to the complex cellular crosstalk regulating implantation [134].

Finally, it is intriguing to consider the development of 3D models of the earliest trophoblast invasion events during human implantation. Recent work co-culturing day 6 human blastocysts or TSC spheroids on confluent Ishikawa endometrial epithelial cell layers demonstrated direct formation of invasive primitive syncytium from the trophectoderm/TSCs for the first time [13]. Recently generated human blastoids (embryo-like structures generated from hESC and TSC) [159], could also be employed in such co-culture models, providing the more accessible high-throughput advantages of TSC spheroids over human blastocysts, but enabling cell–cell signalling between the ICM and polar trophectoderm to be captured. Pairing such work with organ-on-a-chip technologies to increase the cellular complexity of the endometrial layer may also help us better understand the expansion of the cytotrophoblast shell and generation of the first extravillous trophoblast populations during human implantation, in a similar manner to microfluidic models developed to describe the initial interactions between implanting murine embryos and uterine blood vessels [160].

Accurate quantification of trophoblast migration and invasion in improved physiological models provides important data to parameterise in silico models that allow us to understand the dynamic cell–cell relationships involved in trophoblast migration and invasion in new ways [140]. Agent-based computational models of cell migration and invasion, initially developed in the cancer field, enable 3D consideration of the competing forces (i.e. cell–cell attraction, chemotactic influences) on cell behaviour [161], and enable us to look beyond individual cell behaviour to understand the dynamic responses of networks of invading cells. Such modelling remains under-utilised in the placental biology field. However, agent-based models parameterised using time-lapse data of trophoblast migration across endothelial cell monolayers have been used to explore the role of differing mechanical, haemodynamic and chemotactic forces involved in endoarterial trophoblast migration and plug breakdown, shedding new light on the relative importance of different aspects of this process [162]. In turn, lessons learnt from in silico models provide important insight to inform anatomically accurate in vitro experimental design.

### More than trophoblast—the role of mesenchyme in developing anatomically accurate models of placental development

To date, in vitro models of placental development primarily focus on trophoblasts, with little consideration of other villous components, such as mesenchymal cells, which also have important roles in placental structure and function. Indeed, gradients of gene expression across cell types are crucial for normal tissue patterning, and cross-talk between epithelial stem cells and their neighbouring mesenchyme influences stem cell maintenance and renewal, cell fate determination, and organogenesis in multiple tissues, including the intestine and breast [163]. However, the influence of signalling between mesenchymal cells and trophoblast in the placenta, the importance of mesenchyme in maintaining the TSC niche, or how mesenchymal elements may improve or contribute to more physiologically relevant in vitro culture models has received little attention.

The stem cell niche in many epithelial tissues incorporates neighbouring mesenchymal cells, and this may also be the case in the placenta where side-population trophoblasts have been suggested to reside at the mesenchymal border [51]. It is thus likely that mesenchymal cells may have important paracrine effects on both the TSC niche and downstream trophoblast differentiation pathways. In the intestine, mesenchymal cells secrete Wnt2b, a stimulator of canonical Wnt pathways that plays important roles in influencing neighbouring epithelial stem cell maintenance, and in the absence of exogenous Wnt signalling factors, co-culture of mesenchymal cells with intestinal epithelium is sufficient to enable intestinal organoid growth [164, 165]. Analogous canonical Wnt signalling pathways also play important roles in cytotrophoblast and EVT differentiation [17]. Placental mesenchymal cells have also been proposed to stimulate EVT outgrowth via paracrine production of hepatocyte growth factor (HGF), which increases trophoblast migration [166]. Finally, differential expression of Notch receptors and ligands between cytotrophoblasts, syncytiotrophoblast, and mesenchymal cells in the first trimester indicates possible paracrine signalling mechanisms between these cells in developing villi that may promote villus development [167, 168]. In line with this, placental mesenchymal cells enhance proliferation and reduce apoptosis in the HTR8/SVneo trophoblast cell line, an effect attributed to improved mitochondrial function [169]. Whilst the details and regulation of trophoblast-mesenchyme cross-talk and how this relationship may influence villus development are still largely unknown, together these data highlight that identifying the specific factors that placental mesenchymal cells secrete, employing conditioned media from mesenchymal cells, or incorporating placental mesenchymal cells directly into culture models, may have important impacts on trophoblast function, and improve the anatomical and functional accuracy of in vitro studies.

Understanding how mesenchymal-trophoblast cross-talk is altered in pathological pregnancies may also provide important clues to improve trophoblast disease models. A clear example of this is seen in trisomy 21 placentae that feature impaired syncytiotrophoblast fusion and function, where co-culture of trisomy 21 cytotrophoblasts with mesenchymal cells from normal placentae increases cytotrophoblast fusion, an effect partly attributed to the higher Activin-A secretion by the normal mesenchymal cells [170]. Indeed, the paracrine effects of mesenchymal stromal cells (MSCs) on blood vessel growth and function in a range of tissues, including the placenta, are well described, with alterations observed in pregnancy pathologies [171]. However, paracrine implications of other mesenchymal components of the villus core have not been well studied, and this is an important area for future work.

Just as it is important to use trophoblast models that accurately reflect the specific population being studied, due consideration should be given to the relevance of mesenchymal cells employed in co-culture models of placental development. Key transcriptomic differences exist between fetal MSCs that reside within the placenta, and potential contaminating maternal MSCs arising from decidual tissue, and care should be taken to employ culture conditions that enable selection of fetal MSCs or other mesenchymal populations when modelling placental villous development and function [69, 172]. Within the villous core there are also multiple distinct sub-populations of mesenchymal cells, each with distinct transcriptomic and phenotypic attributes [72, 173]. Understanding more about these populations will aid selection of relevant cells of interest for specific in vitro applications. For example, while perivascular mesenchymal populations are appropriate to study placental angiogenesis, those present in the extra-vascular villous stroma adjacent to trophoblasts are likely to be more physiologically relevant to study mesenchymal-trophoblast cross-talk. However, it is important to note that distinct isolated placental mesenchymal populations show rapid phenotypic convergence when cultured in standard medium, and further optimization of tailored conditions to maintain in vivo phenotypes is required [72]. Culture conditions can also have ongoing impacts on placental mesenchymal phenotype and function as, for example, EGM-2 promotes a proliferative MSC phenotype, whilst advanced DMEM/F12 promotes upregulation of contractile markers [69]. Finally, even in organoid models where primary trophoblast proliferation can be sustained, isolated trophoblasts proliferate more slowly in culture than mesenchymal cells, and optimization of cell ratios and methods of combining cells in 3D is likely necessary to prevent mesenchymal overgrowth or achieve a villous architecture that recapitulates the in vivo anatomy.

## Conclusion

Recent advances in the TSC field and adoption of culture media that enable propagation of primary trophoblasts for longer may help enable greater use of primary trophoblast models by increasing accessibility to a wider range of researchers without access to primary tissue, or making isolated cells more practical for use in longer term experiments. Adequate characterisation of cell/tissue viability, purity, and impact of culture conditions, in each model is key to understanding its strengths and limitations with respect to the physiological process being studied. The ongoing development and refinement of 3D and microfluidic culture systems, and future integration of both primary trophoblast and multiple villous cell types into these systems will allow us to study placentation in more physiologically accurate ways, and improve our holistic understanding of placental villous development and function.
